# Impact of burosumab on lower limb alignment in children with X-linked hypophosphatemia

**DOI:** 10.1016/j.jposna.2024.100012

**Published:** 2024-02-28

**Authors:** David B. Frumberg, J. Lawrence Merritt, Angel Chen, Thomas O. Carpenter

**Affiliations:** 1 Department of Orthopaedics and Rehabilitation, Yale University School of Medicine, New Haven, CT, USA; 2 Department of Pediatrics, Yale University School of Medicine, New Haven, CT, USA; 3 Ultragenyx Pharmaceutical Inc, Novato, CA, USA

**Keywords:** Burosumab, Children, Lower limb deformities, Rickets, X-linked hypophosphatemia

## Abstract

**Background:**

Osteotomy and hemiepiphysiodesis are used to treat lower limb deformities in the rare musculoskeletal disease X-linked hypophosphatemia (XLH), but postsurgical complications and malalignment recurrence are possible. This retrospective analysis assessed whether treatment with burosumab, a fully human IgG1 monoclonal antibody to fibroblast growth factor 23 approved for treatment of rickets in XLH, improves lower limb malalignment toward age-specific normal values in children with XLH.

**Methods:**

Children with confirmed XLH received burosumab for 160 weeks in the open-label phase 2 study CL205, or conventional therapy (Pi/D) or burosumab for 64 weeks in the randomized, open-label phase 3 study CL301, with crossover from Pi/D to burosumab through 88 weeks. Full-length, anteroposterior lower limb radiographs were reviewed. The mechanical femoral-tibial angle (MFTA) of lower limbs was measured at baseline and postbaseline. Each MFTA was classified as normal (within 1 standard deviation [SD] of age-specific normal range) or clinically normal (within 2 degrees of normal).

**Results:**

Overall, 116 limbs were included (CL205, *n* = 26; CL301, *n* = 90). Varus or valgus limbs were observed at baseline in 21 (80.8%) limbs in CL205 and in 69 (76.7%) limbs in CL301. In CL205, mean (SD) MFTA decreased from 13.0° (6.7°) at baseline to 5.7° (6.0°) at week 64 and 1.0° (4.8°) at week 160. In CL301, mean (SD) MFTA decreased from 15.5° (13.6°) at baseline to 8.5° (10.0°) at week 64 in the burosumab arm, and in the crossover arm, from 9.2° (10.4°) at week 64 to 6.9° (9.4°) at week 88 (after 22 weeks of burosumab). The proportion of normal or clinically normal limbs increased with burosumab in CL205 (baseline to week 160, 19.2% to 58.3%) and in the CL301 burosumab arm (baseline to week 64, 19.6% to 37.0%) but not in the CL301 crossover arm (week 64-88, 34.1% to 33.3%).

**Conclusions:**

In children with XLH, long-term treatment with burosumab is capable of correcting the MFTA of varus and valgus lower limbs to a neutral alignment without requiring surgical intervention.

**Key Concepts:**

1. Treatment with burosumab led to the correction of lower limb angular deformity to neutral alignment in children with X-linked hypophosphatemia (XLH).
2. Continued treatment with burosumab for at least 1 year appears to have further positive effects on the correction of lower limb angular deformity in children with XLH.
3. Initial treatment with burosumab is indicated in young children with XLH for whom hemiepiphysiodesis is being considered.

**Level of Evidence:**

III

## Introduction

X-linked hypophosphatemia (XLH) is a rare musculoskeletal disease caused by loss-of-function mutations in the *PHEX* gene, resulting in excess production of fibroblast growth factor 23 (FGF23) [1], [2]. The excess plasma FGF23 disrupts renal phosphate reabsorption and 1,25-dihydroxyvitamin D, resulting in hypophosphatemia and defective bone mineralization, followed by rickets, osteomalacia, short stature, bone and joint pain, skeletal abnormalities, and reduced physical function [3], [4], [5].

Lower limb deformities are common in XLH, often consisting of genu valgum or varum, and typically manifest when the child begins walking [6], [7]. Because lower limb malignment persisting into adulthood may contribute to the development of osteoarthritis, fractures, chronic bone and joint pain, and other complications that require surgical realignment [8], early correction is important. Osteotomy to correct lower limb malalignment resulting from XLH is used in adults and in children with severe or complex abnormalities (eg, torsional deformity), but it is invasive and has been associated with postsurgical complications (eg, need for epiphysiodesis, pseudoarthrosis, and fractures) [6], [9], [10], [11]. Guided growth surgery (ie, hemiepiphysiodesis)—a less invasive approach—is beneficial for the gradual correction of coronal plane deformities and may prevent the need for osteotomy in children with XLH; however, multiple procedures may be necessary [7], [9], [12], [13]. Furthermore, hemiepiphysiodesis does not correct the bone at the apex of the deformity and has been associated with the recurrence of lower limb malalignment [6], [14], [15].

Conventional therapy for XLH requires frequent daily dosing of oral phosphate and active vitamin D (Pi/D), which can be burdensome and complicated by safety risks (eg, nephrocalcinosis) and gastrointestinal side effects [16], [17], [18]. Burosumab, a fully human IgG1 monoclonal antibody to FGF23, is approved for the treatment of XLH in children and adults [19], [20]. In open-label phase 2 studies in children aged 1-4 years and 5-12 years with XLH, treatment with burosumab, administered every 2 weeks by subcutaneous injection per study protocols and product labeling, led to improved phosphate metabolism and radiographic rickets healing [16], [17]. In a randomized, active-controlled, open-label phase 3 study in children aged 1-12 years with XLH, 64 weeks of treatment with burosumab, compared with conventional Pi/D therapy, resulted in significantly greater improvements in phosphate metabolism, radiographic rickets healing, and lower limb deformity (ie, genu valgum and genu varum) assessed blindly by independent radiologists [18]. The objective of this retrospective radiographic analysis was to determine whether treatment with burosumab improves lower limb alignment toward age-matched normal values.

## Materials and methods

### Participants

This retrospective analysis included children with XLH who received treatment in the open-label phase 2 study CL205 (ClinicalTrials.gov, NCT02750618) or the randomized, active-controlled, open-label phase 3 study CL301 (ClinicalTrials.gov, NCT02915705) [17], [18]. CL205 was conducted at 3 hospitals in the US. CL301 was conducted at 16 academic health centers in the US, Japan, Canada, the UK, Sweden, South Korea, and Australia.

Principle inclusion criteria were aged 1-4 years (CL205) or 1-12 years (CL301) with radiographic evidence of rickets; serum phosphorus <3.0 mg/dL; serum creatinine in the age-adjusted normal range; *PHEX* mutation or variant of unknown significance confirmed in the child or family member; total Rickets Severity Score ≥2.0 (CL301); and treatment with conventional therapy for ≥6 consecutive months for children aged <3 years or ≥12 months for children aged ≥3 years (CL301). Principal exclusion criteria were hypocalcemia or hypercalcemia; renal ultrasound indicating grade 4 nephrocalcinosis (on a scale of 0-4); [21] planned orthopedic surgery; Tanner stage 4 or 5 (CL301); height ≥50th percentile for age and sex based on country-specific norms (CL301); or plasma intact parathyroid hormone >180 pg/mL (CL301).

The studies were conducted in accordance with the Declaration of Helsinki and the Good Clinical Practice guidelines developed at the International Conference on Harmonization of Technical Requirements for Registration of Pharmaceuticals for Human Use. The institutional review board at each participating site approved the protocol. Parents or guardians provided written informed consent for study participation. Children gave written consent or assent per local guidelines, and an independent committee monitored safety.

### Study treatment

As previously described [17], [18], for 64 weeks, children in both studies received burosumab starting at 0.8 mg/kg every 2 weeks (Q2W), or for study CL301 only, Pi/D conventional therapy titrated per published recommendations (oral phosphate 20-60 mg/kg per day and alfacalcidol 40-60 ng/kg per day or calcitriol 20-30 ng/kg per day) [3], [22]. In both studies, the dose of burosumab was increased to 1.2 mg/kg Q2W if 2 consecutive predose, fasting, serum phosphorus concentrations were <3.2 mg/dL and serum phosphorus had increased by <0.5 mg/dL from baseline in a single measurement. After completion of the 64-week treatment period, children in study CL205 were eligible to continue receiving burosumab in an extension period for a total duration of up to 160 weeks [23], and children in study CL301 were eligible to either continue receiving burosumab or, following a 7-day washout, crossover from Pi/D to burosumab starting at 0.8 mg/kg Q2W for a total duration of up to 124 weeks [24].

### Retrospective analysis of lower limb alignment

The mechanical axis of the limb measured from full-length anteroposterior radiographs is commonly used to determine candidacy for hemiepiphysiodesis or osteotomy for the correction of coronal plane malalignment [7], [25], [26], [27]. The mechanical femoral-tibial angle (MFTA) quantifies the angular mechanical axis deviation. In a quantitative analysis of lower limbs in children with XLH, the majority of identified abnormalities were in the femoral shaft, and the femoral-tibial angle was significantly different between children with genu varum or valgum compared with aligned lower limbs or with normal controls [28]. Age group-specific MFTA normal ranges for children aged 1-18 years have been determined based on anteroposterior radiographs [29].

**Figure 1:**
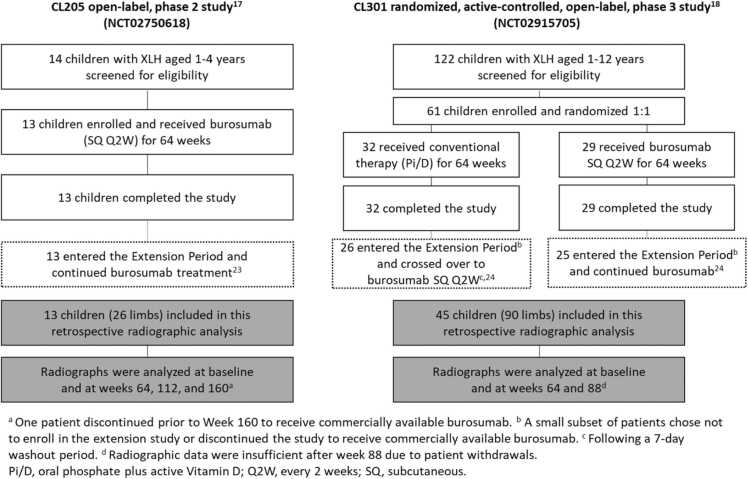
Patient disposition in the CL205 and CL301 studies, extension periods, and the retrospective radiographic analysis. XLH, X-linked hypophosphatemia.

In this retrospective analysis, full-length anteroposterior lower limb radiographs were taken from children in CL205 and CL301 who were standing with the patella facing forward. Two patients were excluded because standard radiographic techniques were not used. The radiographs were reviewed in a blinded fashion by an orthopedic surgeon (DF). The review of radiographs was blind to treatment for study CL301 but not CL205, which was open label. The MFTA of each lower limb was measured from the center of the femoral head to the center of the knee and from the center of the knee to the center of the ankle using Bone Ninja (International Center for Limb Lengthening, Baltimore, MD) [27]. Radiographs from study CL205 were assessed at baseline and at weeks 64, 112, and 160. Radiographs from study CL301 were assessed at baseline and at weeks 64 and 88; radiographic data were insufficient after week 88 because patients left the study to receive commercially available burosumab (Fig. 1). Limbs from children in CL301 with a history of osteotomy or hemiepiphysiodesis were not included; orthopedic surgery history was not collected for children in CL205. Limb concordance or discordance within patients was not an objective of this analysis, and thus no results are reported.

The primary outcome of the analysis was the change over time in MFTA. At all study time points, MFTA measurements, expressed in degrees, were assessed for individual lower limbs. MFTA data were also expressed as the percentage change from baseline and as the change from baseline in degrees.

**Figure 2:**
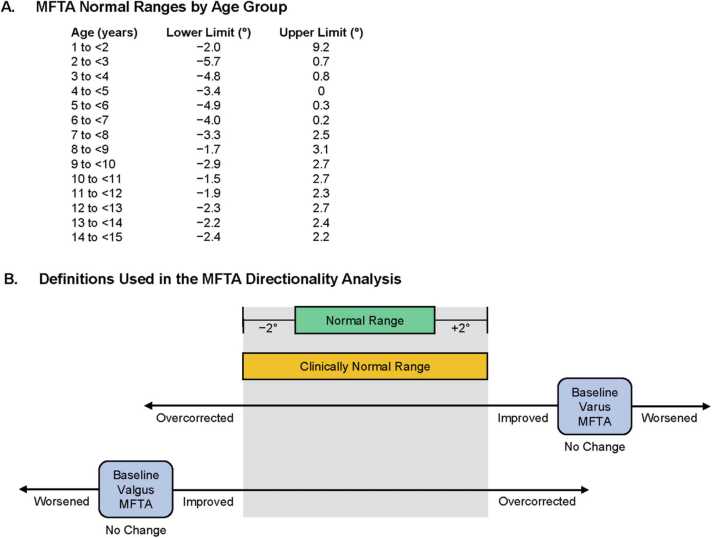
Classification of lower limb MFTA. (A) MFTA normal ranges by age group. The lower and upper limits of the MFTA normal range for each age group were calculated using the mean ± 1 SD reported by Sabharwal and Zhao [29]. (B) Definitions used for classification of lower limb MFTA. At each time point, the MFTA measured for each limb was compared with the normal range of the appropriate age group, with the normal range adjusted for new age at each radiographic measurement. Limbs at each time point were categorized using the following definitions: *Normal* (an MFTA within 1 SD above or below the age group-specific mean normal hip-knee-ankle angle values reported by Sabharwal and Zhao [29]); *Clinically Normal* (an MFTA not achieving normal status but was no more than 2 degrees above or no less than 2 degrees below the age group-specific normal range); *Valgus* (an MFTA below the age group-specific clinically normal range); *Varus* (an MFTA above the age group-specific clinically normal range); *Improved* (a valgus MFTA that increased from baseline but remained below the clinically normal range, or a varus MFTA that decreased from baseline but remained above the clinically normal range); *No Change* (a valgus or varus MFTA that did not change from baseline); *Overcorrected* (a valgus MFTA that increased from baseline to above the upper clinically normal age group-specific range, or a varus MFTA that decreased from baseline to below the lower clinically normal age group-specific range); or worsened (a valgus MFTA that further decreased from the baseline value, or a varus MFTA that further increased from the baseline value). MFTA, mechanical femorotibial angle; SD, standard deviation.

An additional outcome of the analysis was the proportion of lower limbs with MFTA classified as normal, clinically normal, valgus, or varus each study time point. A normal MFTA was defined as any MFTA within 1 standard deviation (SD) above or below the age group-specific normal MFTA ranges reported by Sabharwal et al. [29] (Fig. 2A). A clinically normal MFTA (ie, a neutral MFTA) was defined as any MFTA not meeting normal criteria but that was no more than 2 degrees below or above the age group-specific normal range (Fig. 2B). Thus, limbs classified as “clinically normal” were exclusive of those classified as “normal.” A valgus MFTA was defined as any MFTA below the age group-specific clinically normal range. A varus MFTA was defined as any MFTA above the age group-specific clinically normal range. Lower limbs that did not meet normal or clinically normal MFTA criteria (ie, valgus or varus) at a postbaseline assessment were classified based on their change from baseline MFTA (Fig. 2B). An improved MFTA was defined as a valgus MFTA that increased from baseline but remained below the clinically normal range, or a varus MFTA that decreased from baseline but remained above the clinically normal range. An MFTA with no change was defined as a valgus or varus MFTA that did not change from baseline. An overcorrected MFTA was defined as a valgus MFTA that increased to above the upper clinically normal age group-specific range or a varus MFTA that decreased to below the lower clinically normal age group-specific range. A worsened MFTA was defined as a valgus MFTA that further decreased from the baseline value or a varus MFTA that further increased from the baseline value. Data were expressed as the proportions of limbs meeting a classification.

### Statistical analysis

Data were described descriptively and were summarized using means with SDs.

## Results

### Baseline lower limb alignment

The CL205 and CL301 study populations have been described [17], [18], [23], [24]. In total, 116 limbs were assessed in this retrospective analysis, including 26 limbs from all 13 children in study CL205 and 90 limbs from 45 children in study CL301 (burosumab, *n* = 46 limbs; Pi/D, *n* = 44 limbs). Radiographic data were available for 24 limbs at week 88 of study CL301 following the crossover from Pi/D to burosumab at week 66.

**Figure 3:**
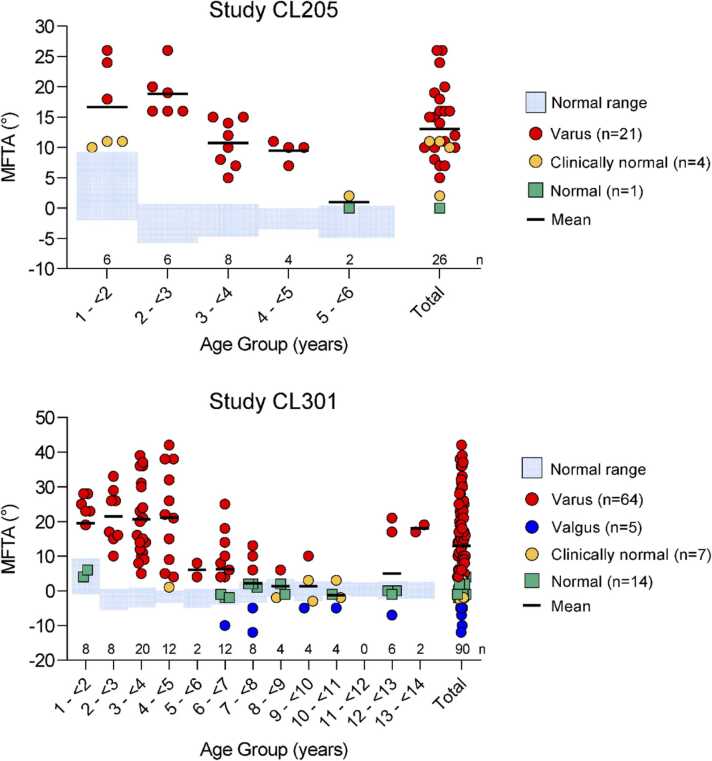
Baseline MFTA classification by age group. Each circle or square represents an individual lower limb MFTA. MFTA, mechanical femorotibial angle.

At baseline, 21 of 26 (80.8%) limbs in study CL205 were varus, four (15.4%) were clinically normal, and one (3.8%) was normal (Table 1, Fig. 3). At baseline, 64 of 90 (71.1%) limbs in study CL301 were varus (burosumab, *n* = 35; Pi/D, *n* = 29), 14 (15.6%) were normal (burosumab, *n* = 6; Pi/D, *n* = 8), seven (7.8%) were clinically normal (burosumab, *n* = 3; Pi/D, *n* = 4), and five (5.6%) were valgus (burosumab, *n* = 2; Pi/D, *n* = 3). Of the 14 normal limbs, eight were from 4 patients with both limbs measuring normal, and the remaining six were from separate patients.

**Table 1:** Demographics and baseline characteristics.

		CL301		
	CL205 (*n* = 26)	Burosumab (*n* = 46)	Crossover (*n* = 44)	Total (*N* = 90)
Mean (SD) age, years	2.9 (1.1)	5.1 (3.1)	5.9 (3.4)	5.5 (3.2)
**Sex, *n (%)***				
Male	18 (69.2)	14 (30.4)	20 (45.5)	34 (37.8)
Female	3 (30.8)	32 (69.6)	24 (54.6)	56 (62.2)
**Lower limb MFTA alignment, *n (%)***				
Varus	21 (80.8)	35 (76.1)	29 (65.9)	64 (71.1)
Valgus	0	2 (4.3)	3 (6.8)	5 (5.6)
Normal	1 (3.8)	6 (13.0)	8 (18.2)	14 (15.6)
Clinically normal	4 (15.4)	3 (6.5)	4 (9.1)	7 (7.8)
**MFTA,° degrees**				
*Mean (SD)*				
Baseline	13.0 (6.7)	15.5 (13.6)	10.3 (11.7)	13.0 (12.9)
1 to <2 y (*n* = 6^a^; 8^b^)	16.7 (7.1)	13.0 (9.4)	26.0 (2.5)	19.5 (9.4)
2 to <3 y (*n* = 6^a^; 8^b^)	18.8 (3.9)	18.3 (6.4)	31.0 (2.8)	21.5 (8.1)
3 to <4 y ( *n* = 8^a^; 20^b^)	10.8 (3.9)	26.7 (10.5)	14.4 (7.5)	20.6 (10.9)
4 to <5 y ( *n* = 4^a^; 12^b^)	9.5 (1.7)	26.7 (17.3)	15.5 (9.2)	21.1 (14.4)
5 to <6 y ( *n* = 2^a^; 2^b^)	1.0 (1.4)	—	6.0 (2.8)	6.0 (2.8)
6 to <7 y ( *n* = 0^a^; 12^b^)	—	6.6 (10.9)	5.3 (8.0)	6.2 (9.7)
7 to <8 y ( *n* = 0^a^; 8^b^)	—	7.5 (5.2)	−3.3 (6.7)	2.1 (8.0)
8 to <9 y ( *n* = 0^a^; 4^b^)	—	4.0 (2.8)	−1.5 (0.7)	1.3 (3.6)
9 to <10 y ( *n* = 0^a^; 4^b^)	—	—	1.3 (6.8)	1.3 (6.8)
10 to <11 y ( *n* = 0^a^; 4^b^)	—	−1.3 (3.3)	—	−1.3 (3.3)
11 to <12 y ( *n* = 0^a^; 0^b^)	—	—	—	—
12 to <13 y ( *n* = 0^a^; 6^b^)	—	—	5.0 (11.2)	5.0 (11.2)
13 to <14 y ( *n* = 0^a^; 2^b^)	—	18.0 (1.4)	—	18.0 (1.4)
*Median (Q1, Q3)*				
Baseline	11.5 (10.0, 16.0)	14.5 (4.0, 25.0)	9.5 (0.0, 21.0)	10.5 (3.0, 23.0)
1 to <2 y ( *n* = 6^a^; 8^b^)	14.5 (11.0, 24.0)	12.5 (5.0, 21.0)	26.5 (24.0, 28.0)	23.0 (12.5, 26.5)
2 to <3 y ( *n* = 6^a^; 8^b^)	17.5 (16.0, 20.0)	16.5 (15.0, 26.0)	31.0 (29.0, 33.0)	21.5 (15.5, 27.5)
3 to <4 y ( *n* = 8^a^; 20^b^)	11.0 (7.5, 14.5)	27.5 (15.0, 36.0)	12.5 (9.0, 20.0)	18.0 (11.5, 30.5)
4 to <5 y ( *n* = 4^a^; 12^b^)	10.0 (8.5, 10.5)	35.0 (9.0, 38.0)	18.0 (5.0, 22.0)	21.5 (7.0, 35.0)
5 to <6 y ( *n* = 2^a^; 2^b^)	1.0 (0.0, 2.0)	—	6.0 (4.0, 8.0)	6.0 (4.0, 8.0)
6 to <7 y ( *n* = 0^a^; 12^b^)	—	5.0 (1.0, 13.0)	4.5 (−1.5, 12.0)	5.0 (−1.5, 12.0)
7 to <8 y ( *n* = 0^a^; 8^b^)	—	8.0 (3.5, 11.5)	−1.5 (−8.5, 2.0)	2.0 (−2.0, 8.0)
8 to <9 y ( *n* = 0^a^; 4^b^)	—	4.0 (2.0, 6.0)	−1.5(−2.0, −1.0)	0.5 (−1.5, 4.0)
9 to <10 y ( *n* = 0^a^; 4^b^)	—	—	0.0 (−4.0, 6.5)	0.0 (−4.0, 6.5)
10 to <11 y ( *n* = 0^a^; 4^b^)	—	−1.5 (−3.5, 1.0)	—	−1.5 (−3.5, 1.0)
11 to <12 y ( *n* = 0^a^; 0^b^)	—	—	—	—
12 to <13 y ( *n* = 0^a^; 6^b^)	—	—	0.0 (−1.0, 17.0)	0.0 (−1.0, 17.0)
13 to <14 y ( *n* = 0^a^; 2^b^)	—	18.0 (17.0, 19.0)	—	18.0 (17.0, 19.0)

^a^ CL205. ^b^ CL301. MFTA, mechanical femorotibial angle; SD, standard deviation; Q1, first quartile; Q3, third quartile.

The mean MFTA at baseline in both studies tended to be greater among younger age groups than in older age groups (Table 1, Fig. 3). The overall baseline mean (SD) MFTA was 13.0° (6.7°) in study CL205 and 13.0° (12.9°) in study CL301.

### Change in lower limb malalignment with burosumab

**Figure 4:**
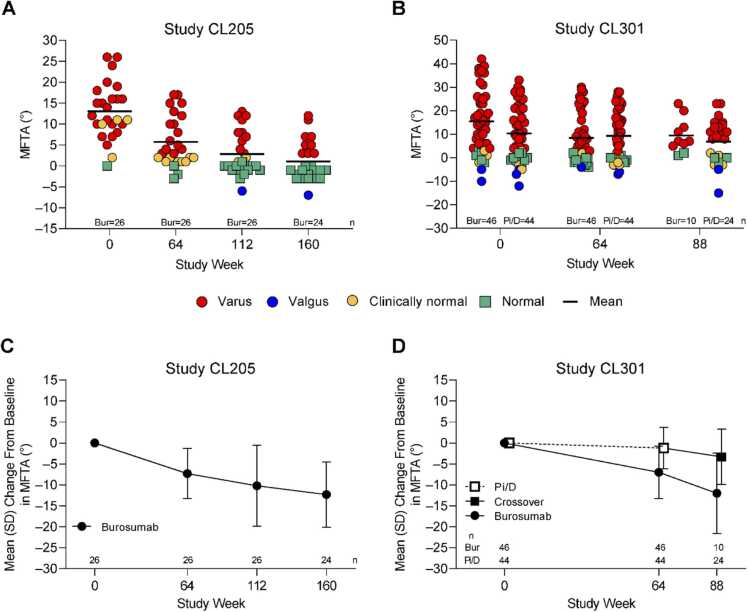
Change in MFTA. (A) Summary of MFTA at each study time point in study CL205. (B) Summary of MFTA at each study time point in study CL301. (C) Mean (SD) change from baseline in MFTA (degrees) at each study time point in study CL205. (D) Mean (SD) change from baseline in MFTA (degrees) at each study time point in study CL301. MFTA, mechanical femorotibial angle; SD, standard deviation.

**Figure 5:**
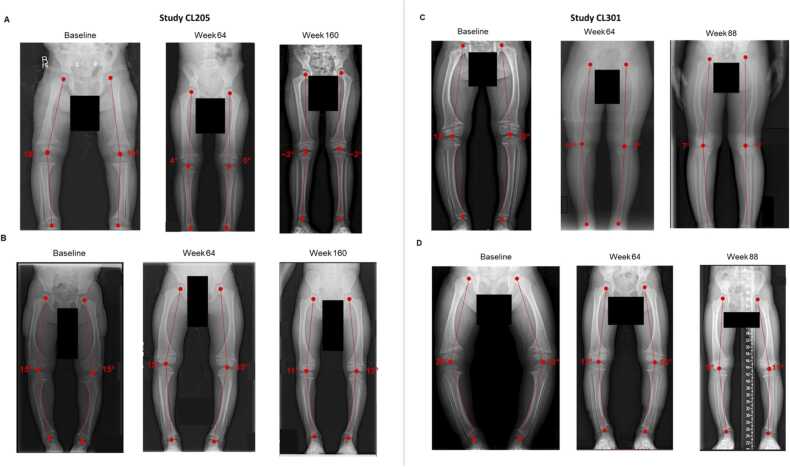
Lower limb radiographs. (A) Study CL205 lower limb radiographs from a 1.6-year-old male at baseline and after 64 and 160 weeks of treatment with burosumab. (B) Study CL205 lower limb radiographs from a 3.7-year-old male at baseline and after 64 and 160 weeks of treatment with burosumab. (C) Study CL301 lower limb radiographs from a 7.2-year-old female at baseline and after 64 and 88 weeks of treatment with burosumab. (D) Study CL301 lower limb radiographs from a 2.1-year-old female at baseline who had 64 weeks of conventional therapy (Pi/D) and then crossed over to burosumab through 88 weeks of treatment. Mechanical femorotibial angle measurements are illustrated in red.

Change over time in MFTA is summarized by MFTA alignment category for study CL205 in Fig. 4A and for study CL301 in Fig. 4B. In study CL205, mean (SD) MFTA decreased from 13.0° (6.7°) at baseline to 5.7° (6.0°) after 64 weeks of treatment with burosumab (change from baseline, −7.3°; Fig. 4C). After 160 weeks of treatment with burosumab, MFTA further decreased to 1.0° (4.8°) (change from baseline, −12.3°). In the study CL301 burosumab arm, MFTA decreased from 15.5° (13.6°) at baseline to 8.5° (10.0°) after 64 weeks of treatment (change from baseline, −7.0°) and to 9.5° (7.3°) after 88 weeks of treatment (change from baseline, −12.0°; Fig. 4D). In the study CL301 Pi/D arm, MFTA decreased from 10.3° (11.7°) at baseline to 9.2° (10.4°) after 64 weeks of treatment (change from baseline, −1.2°). At week 88, 22 weeks after crossover from Pi/D to burosumab, there was a further decrease to 6.9° (9.4°) (change from baseline, −3.3°). Examples of lower leg radiographs showing improvement or correction of lower limb malalignment with burosumab in studies CL205 and CL301 are included in Fig. 5.

In study CL205, the proportion of limbs meeting the criteria for normal or clinically normal (ie, those with neutral alignment) increased from 19.2% at baseline (normal, 3.8%; clinically normal, 15.4%) to 42.3% after 64 weeks of burosumab (normal, 11.5%; clinically normal, 30.8%), and to 58.3% after 160 weeks of treatment (normal, 58.3%; clinically normal, 0%; Supplemental Fig. 1A, Supplemental Table 1). In the study CL301 burosumab arm, the proportion of limbs with neutral alignment increased from 19.6% at baseline (normal, 13.0%; clinically normal, 6.5%) to 37.0% after 64 weeks of burosumab (normal, 32.6%; clinically normal, 4.3%), and to 20.0% after 88 weeks of burosumab (normal, 20.0%; clinically normal, 0%; Supplemental Fig. 1B, Supplemental Table 2). In the CL301 Pi/D arm, the proportion of limbs with neutral alignment increased from 27.3% at baseline (normal, 18.2%; clinically normal, 9.1%) to 34.1% after 64 weeks of treatment with Pi/D (normal, 22.7%; clinically normal, 11.4%), and to 33.3% at week 88, 22 weeks after crossover from Pi/D to burosumab (normal, 16.7%; clinically normal, 16.7%; Supplemental Fig. 1B, Supplemental Table 2).

Among limbs in study CL205 that did not correct to neutral alignment with burosumab (ie, limbs that remained valgus or varus), improvement occurred in 73.3% of limbs after 64 weeks of treatment and in 100% of limbs after 160 weeks of treatment (Supplemental Fig. 2A, Supplemental Table 1). Among limbs in study CL301 that remained valgus or varus with burosumab, improvement occurred in 93.1% of limbs after 64 weeks of burosumab and in 56.3% at week 88, 22 weeks after crossover from Pi/D to burosumab (Supplemental Fig. 2B, Supplemental Table 2). Among the limbs that remained valgus or varus in study CL205, mean (SD) MFTA decreased from 15.8° (6.3°) at baseline to 9.5° (5.2°) after 64 weeks of burosumab and to 6.2° (3.4°) after 160 weeks of burosumab (Supplemental Fig. 2C). Likewise, among limbs remaining valgus or varus in study CL301, MFTA in the burosumab arm decreased from 21.9° (11.8°) at baseline to 13.6° (9.2°) after 64 weeks and to 11.5° (6.8°) after 88 weeks (Supplementary Fig. 2D). Among limbs remaining valgus or varus in the study CL301 Pi/D arm of study CL301, MFTA decreased from 14.1° (11.9°) at baseline to 10.7° (9.4°) at week 88, 22 weeks after crossover from Pi/D to burosumab.

In study CL205, a child with varus limbs at baseline had 1 limb correct to neutral alignment and 1 limb overcorrect to valgus alignment after 112 weeks of burosumab, while another child with varus limbs at baseline had both limbs temporarily worsen before improving (Supplemental Fig. 2A, Supplemental Table 1). In study CL301, the MFTA worsened from baseline to week 64 in 11 limbs from 7 children (burosumab, *n* = 1; Pi/D, *n* = 10) and 22 weeks after crossover from Pi/D to burosumab in 4 limbs from 3 children. Changes in phosphorus metabolism and serum alkaline phosphatase (ALP) did not appear to explain worsened MFTA (see below).

In studies CL205 and CL301, treatment with burosumab led to mean increases from baseline in serum phosphorus to concentrations at or above the lower limit of normal and mean decreases in ALP to concentrations at or below the upper limit of normal (Supplemental Fig. 3A-D). In both studies, there were no discernable differences in serum concentrations of phosphorus or ALP between children with normal limbs that corrected to neutral alignment compared with children whose limbs remained varus or valgus during treatment. The apparent increase in mean ALP from week 64 to week 88 in the study CL301 burosumab arm is the result of very high levels (205% of ULN) in 1 of the 6 children.

## Discussion

Treatment with burosumab improves phosphate metabolism, as well as rickets healing and associated limb deformity assessed using the Radiographic Global Impression of Change, which measures the relative change in severity in the wrist, knee, and leg [16], [17], [18], [30]. This large analysis of 116 lower limbs from 58 children with XLH investigated the effect of treatment with burosumab on the MFTA, which is often used to determine whether a malaligned lower limb is a candidate for surgical correction [7], [25], [26], [27]. The improvements in mean MFTA associated with burosumab were observed only after treatment for 64 weeks (mean decrease of 7.3° in study CL205 and 7.0° in study CL301) and were ongoing at 160 weeks (mean decrease of 12.3° in study CL205), indicating that sustained amelioration of hypophosphatemia for at least 1 year is needed for measurable, favorable changes at the physes of malaligned lower limbs in children with XLH.

Given the invasiveness of osteotomy and hemiepiphysiodesis, as well as the potential for postsurgical complications and recurrence [6], [7], [9], [10], [11], [12], [13], [14], [15], it is noteworthy that treatment with burosumab corrected lower limb malignment to a neutral alignment (ie, not requiring orthopedic surgery) in a large proportion of limbs after 64 weeks of treatment (42.3% of limbs in study CL205; 37.0% of limbs in study CL301) and continuing up to 160 weeks of treatment (58.3% in study CL205). Treatment with burosumab also improved the MFTA among lower limbs that remained valgus at postbaseline, with a mean decrease of 6.3° after 64 weeks in study CL205, a mean decrease of 9.6° after 160 weeks in study CL205, a mean decrease of 8.3° after 64 weeks in study CL301, and a mean decrease of 3.4° after 22 weeks of burosumab crossover in study CL301.

In contrast with our observations of correction of MFTA alignment with burosumab, Mindler et al. [31] reported persistent axial and coronal plane deformities, as well as 1 case of new onset valgus deformity following 1 year of burosumab in a small case series (*n* = 14 pediatric limbs). Differences in this study population (eg, no prior or current orthopedic surgery history in CL301), study design, and methods versus those of Mindler et al. preclude direct comparison of findings. Importantly, *de novo* varus or valgus deformities were not observed during studies CL205 and CL301, and only 1 of 11 limbs with worsened MFTA occurred during treatment with burosumab in CL301.

Consistent with prior observations [16], [17], [18], serum phosphorus metabolism and ALP among the children included in this analysis were improved by treatment with burosumab, including after crossover from Pi/D to burosumab in study CL301. However, at later time points, small sample sizes due to patient discontinuation for commercially available burosumab prevented the discernment of differences in serum phosphorus and ALP between subgroups of lower limbs based on the extent of MFTA correction. The importance of other disease and patient factors (eg, age at treatment initiation) on the physeal changes that occur during treatment with burosumab in children with XLH is unclear. Treatment with burosumab improves rickets healing, compared with Pi/D, in both younger and older children [32]. In preclinical studies in *Hyp* mice, inhibition of FGF23 increased femoral growth, narrowing of the distal femoral metaphysis, and reorganization of the growth plate area characterized by enlargement of the proliferative zone [33], [34].

This analysis was strengthened by the overall large number of limbs included in the clinical studies, the extended time period of burosumab treatment, particularly for study CL205, and the randomized, controlled design of study CL301. Limitations of this analysis included the lack of follow-up data on orthopedic surgery following study completion, the open-label design and lack of a placebo control group in the CL205 study, and patient dropout after week 64 in study CL301 due to the availability of commercial burosumab. Additionally, because only 1 orthopedic surgeon reviewed the radiographs, there were no intra- and interobserver reliability measurements. However, the images were read in a blinded fashion, and the MFTA is highly reproducible when the patient is standing, patella is facing forward, and the hip and ankle positions are known [35], [36].

In conclusion, by ameliorating hypophosphatemia in patients with XLH, burosumab is capable of correcting the MFTA of varus and valgus lower limbs to a neutral alignment, often avoiding the need for surgical intervention. Sustained treatment with burosumab for at least 1 year appears to be necessary for substantial correction of lower limb malalignment. Further investigation is needed to characterize the changes at the distal femoral physis in children with XLH during treatment with burosumab. Future investigations should also include an assessment of the effect of burosumab and initiation of treatment at an earlier age on sagittal and axial plane alignment and limb concordance in children with XLH, as well as genotype-phenotype associations of the *PHEX* gene in predicting improvement with burosumab treatment.

## Additional links

• Burosumab Therapy in Children with X-Linked Hypophosphatemia.
• Burosumab Improves Lower Limb Alignment in Children with X-Linked Hypophosphatemia.

## Author contributions

**Frumberg David B.:** Conceptualization, Data curation, Formal analysis, Methodology, Writing – review & editing, Investigation, Visualization, Writing – original draft. **Merritt II J. Lawrence:** Conceptualization, Visualization, Writing – review & editing, Writing – original draft. **Chen Angel:** Formal analysis, Writing – review & editing, Conceptualization, Visualization. **Carpenter Thomas O.:** Conceptualization, Supervision, Writing – review & editing, Methodology, Visualization, Investigation, Writing – original draft.

## Funding

This study was sponsored and funded by Ultragenyx Pharmaceutical Inc. in partnership with Kyowa Kirin, Inc.

## Declaration of competing interests

The authors declare the following financial interests/personal relationships which may be considered as potential competing interests: David B. Frumberg reports that financial support was provided by Ultragenyx Pharmaceutical Inc. David B. Frumberg reports that financial support was provided by Orthofix Medical Inc. David B. Frumberg reports that financial support was provided by OrthoPediatrics. J. Lawrence Merritt II reports that financial support was provided by Ultragenyx Pharmaceutical Inc. Angel Chen reports that financial support was provided by Ultragenyx Pharmaceutical Inc. Thomas O. Carpenter reports that financial support was provided by Ultragenyx Pharmaceutical Inc. Thomas O. Carpenter reports that financial support was provided by Kyowa Kirin, Inc. David B. Frumberg reports a relationship with Ultragenyx Pharmaceutical Inc. that includes consulting or advisory. David B. Frumberg reports a relationship with Orthofix Medical Inc. that includes consulting or advisory. David B. Frumberg reports a relationship with OrthoPediatrics that includes consulting or advisory. J. Lawrence Merritt II reports a relationship with Ultragenyx Pharmaceutical Inc. that includes employment and equity or stocks. Angel Chen reports a relationship with Ultragenyx Pharmaceutical Inc. that includes employment and equity or stocks. Thomas O. Carpenter reports a relationship with Ultragenyx Pharmaceutical Inc. that includes consulting or advisory and funding grants. Thomas O. Carpenter reports a relationship with Kyowa Kirin, Inc. that includes consulting or advisory. If there are other authors, they declare that they have no known competing financial interests or personal relationships that could have appeared to influence the work reported in this paper.

## Footnotes

Supplementary material associated with this article can be found in the online version at doi:10.1016/j.jposna.2024.100012.
