# Supplementary material for: Impact of burosumab on lower limb alignment in children with X-linked hypophosphatemia
Source: J Pediatr Soc North Am. 2024 Feb 28;6:100012. doi: 10.1016/j.jposna.2024.100012 (PMC12088215; doi:10.1016/j.jposna.2024.100012)
Supplement: Supplementary file 1 — Supplementary material [file mmc1.docx]

**Supplemental Table 1. Summary of Lower Limb MFTA Classification in Study CL205**

|  | **Baseline (n=26)** | **Week 64 (n=26)** | **Week 112 (n=26)** | **Week 160 (n=24)** |
| --- | --- | --- | --- | --- |
| Normal, n (%) | 1 (3.8) | 3 (11.5) | 13 (50) | 14 (58.3) |
| Clinically normal, n (%) | 4 (15.4) | 8 (30.8) | 2 (7.7) | 0 |
| Varus, n (%) | 21 (80.8) | 15 (57.7) | 10 (38.5) | 9 (37.5) |
| Improved | — | 11 (42.3) | 9 (34.6) | 9 (37.5) |
| No change | — | 2 (7.7) | 1 (3.8) | 0 |
| Worsened | — | 2 (7.7) | 0 | 0 |
| Overcorrected | — | 0 | 0 | 0 |
| Valgus, n (%) | 0 | 0 | 1 (3.8) | 1 (3.8) |
| Improved | — | 0 | 0 | 0 |
| No change | — | 0 | 0 | 0 |
| Worsened | — | 0 | 0 | 0 |
| Overcorrected | — | 0 | 1 (3.8) | 1 (3.8) |

MFTA, mechanical femorotibial angle. *Normal*: an MFTA within one SD above or below the age group–specific mean normal hip-knee-ankle angle value reported by Sabharwal et al^28^. *Clinically normal*: an MFTA no more than 2 degrees above or no less than 2 degrees below the age group–specific normal range. *Valgus*: an MFTA below the age group–specific clinically normal range. *Varus*: MFTA above the age group–specific clinically normal range. *Improved*: a valgus MFTA that increased from baseline but remained below the clinically normal range, or a varus MFTA that decreased from baseline but remained above the clinically normal range. *No change*: a valgus or varus MFTA that did not change from baseline. *Overcorrected*: a valgus MFTA that increased from baseline to above the upper clinically normal age group–specific range, or a varus MFTA that decreased from baseline to below the lower clinically normal age group–specific range. *Worsened*: a valgus MFTA that further decreased from the baseline value, or a varus MFTA that further increased from the baseline value.

**Supplemental Table 2. Summary of Lower Limb MFTA Classification in Study CL301**

|  | **Baseline (n=90)** | |  | **Week 64 (n=90)** | |  | **Week 88 (n=34)** | |
| --- | --- | --- | --- | --- | --- | --- | --- | --- |
|  | **Burosumab (n=46)** | **Pi/D (n=44)** |  | **Burosumab (n=46)** | **Pi/D (n=44)** |  | **Burosumab (n=10)** | **Crossover (n=24)** |
| Normal, n (%) | 6 (13.0) | 8 (18.2) |  | 15 (32.6) | 10 (22.7) |  | 2 (20) | 4 (16.7) |
| Clinically normal, n (%) | 3 (6.5) | 4 (9.1) |  | 2 (4.3) | 5 (11.4) |  | 0 | 4 (16.7) |
| Varus, n (%) | 35 (76.1) | 29 (65.9) |  | 28 (60.9) | 27 (61.4) |  | 8 (80) | 14 (58.3) |
| Improved | — | — |  | 26 (56.5) | 16 (36.4) |  | 7 (70) | 9 (37.5) |
| No change | — | — |  | 1 (2.2) | 2 (4.5) |  | 1 (10) | 2 (8.3) |
| Worsened | — | — |  | 1 (2.2) | 9 (20.5) |  | 0 | 3 (12.5) |
| Overcorrected | — | — |  | 0 | 0 |  | 0 | 0 |
| Valgus, n (%) | 2 (4.3) | 3 (6.8) |  | 1 (2.2) | 2 (4.5) |  | 0 | 2 (8.3) |
| Improved | — | — |  | 1 (2.2) | 1 (2.3) |  | 0 | 0 |
| No change | — | — |  | 0 | 0 |  | 0 | 1 (4.2) |
| Worsened | — | — |  | 0 | 1 (2.3) |  | 0 | 1 (4.2) |
| Overcorrected | — | — |  | 0 | 0 |  | 0 | 0 |

MFTA, machanical femorotibial angle. *Normal*: an MFTA within one SD above or below the age group–specific mean normal hip-knee-ankle angle value reported by Sabharwal et al^28^. *Clinically normal*: an MFTA no more than 2 degrees above or no less than 2 degrees below the age group–specific normal range. *Valgus*: an MFTA below the age group–specific clinically normal range. *Varus*: MFTA above the age group–specific clinically normal range. *Improved*: a valgus MFTA that increased from baseline but remained below the clinically normal range, or a varus MFTA that decreased from baseline but remained above the clinically normal range. *No change*: a valgus or varus MFTA that did not change from baseline. *Overcorrected*: a valgus MFTA that increased from baseline to above the upper clinically normal age group–specific range, or a varus MFTA that decreased from baseline to below the lower clinically normal age group–specific range. *Worsened*: a valgus MFTA that further decreased from the baseline value, or a varus MFTA that further increased from the baseline value


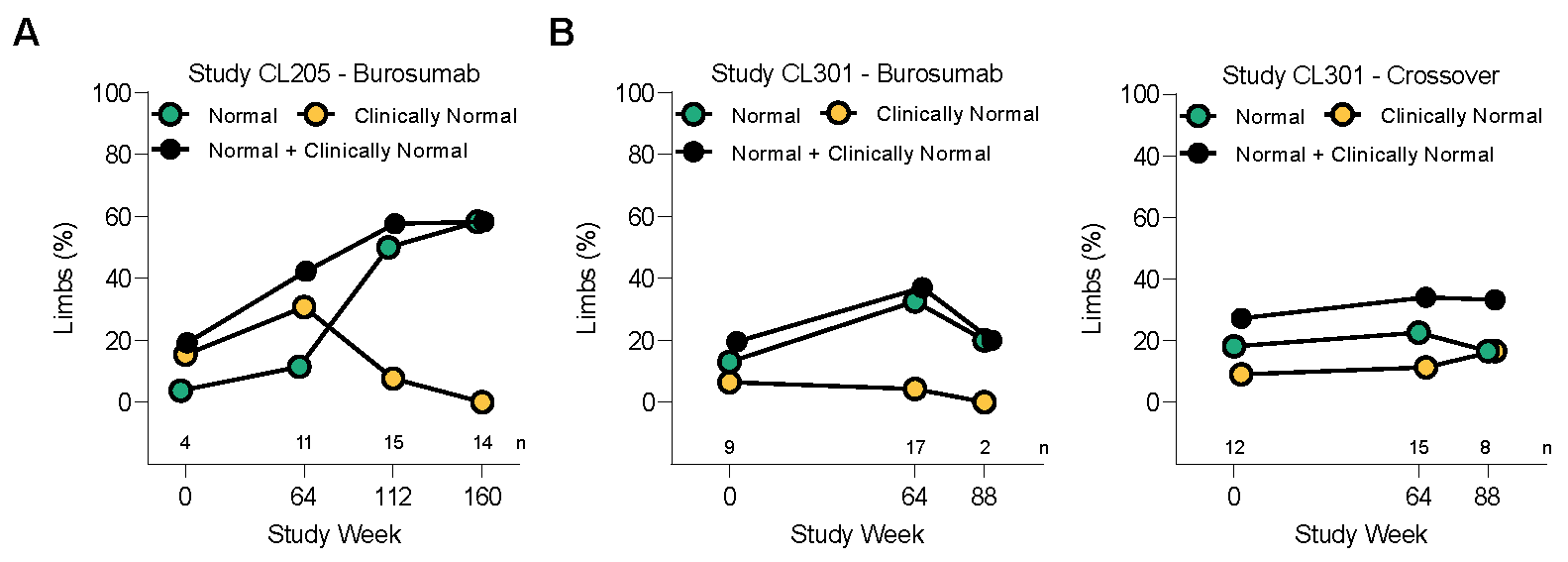


**Supplemental Figure 1.** Proportion of limbs meeting the criteria for normal or clinically normal at each study time point in study CL205 (**A**) and in study CL301 (**B**). n = the number of limbs meeting the criteria for normal or clinically normal.


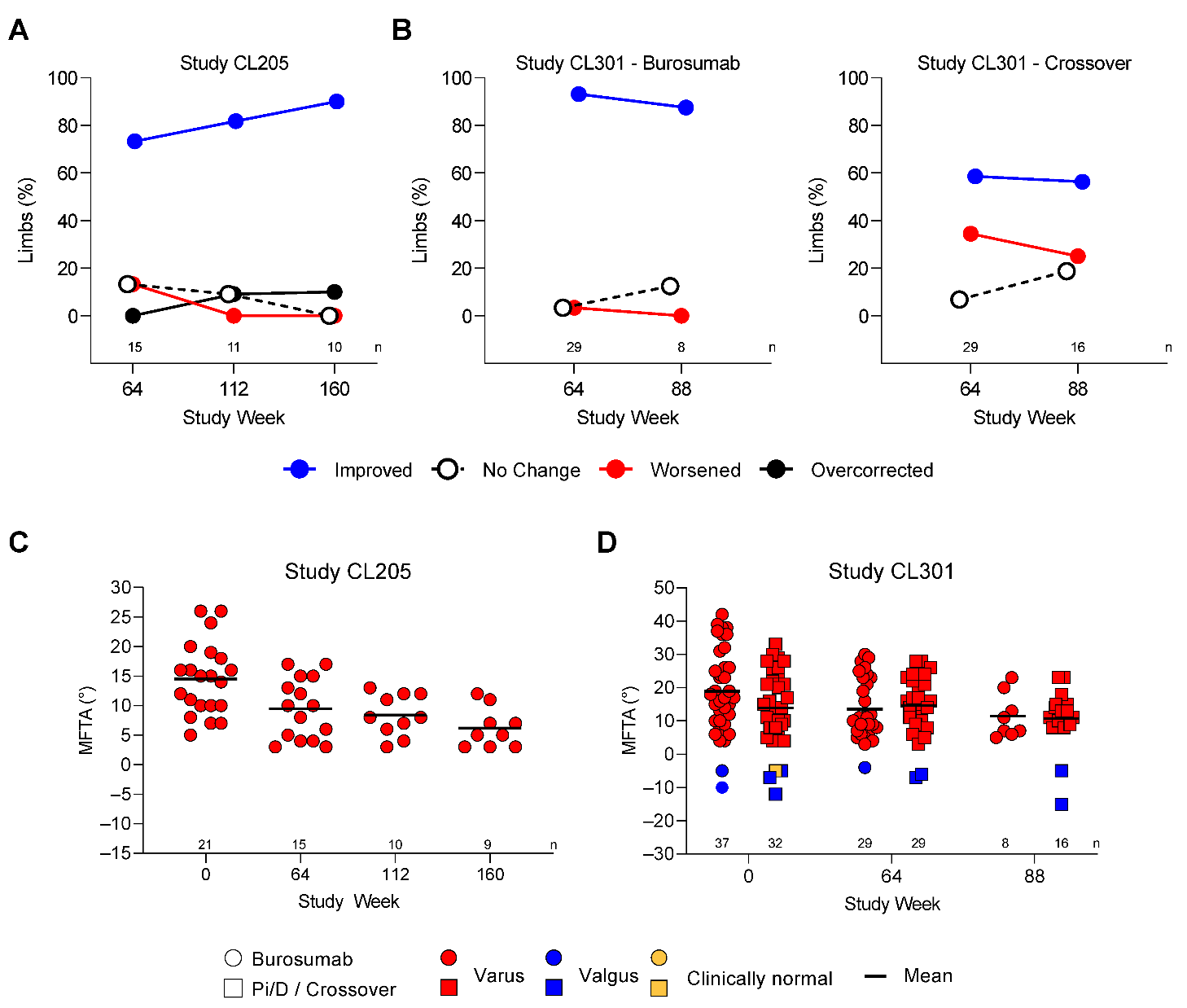


**Supplemental Figure 2**. Improvements among limbs remaining valgus or varus. **A**, Proportions of limbs remaining valgus/varus that improved, had no change, worsened, or were overcorrected at each study time point in Study CL205. **B**, Proportions of limbs remaining valgus/varus that improved, had no change, worsened, or were overcorrected at each study time point in Study CL301. **C**, Summary of MFTA among limbs remaining valgus/varus at each study time point in Study CL205. **D**, Summary of MFTA among limbs remaining valgus/varus at each study time point in Study CL301.


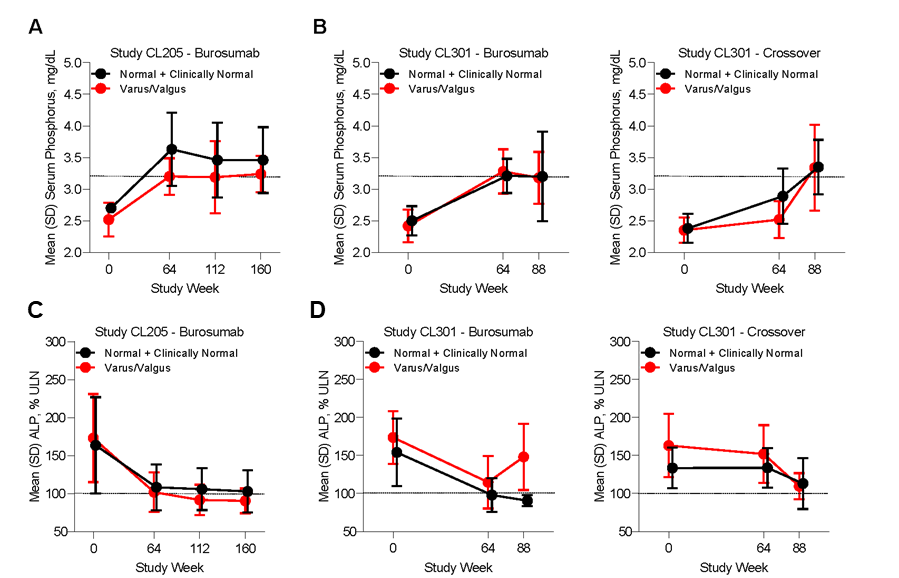


**Supplemental Figure 3.** Subgroup assessment of serum phosphorus and alkaline phosphatase. Change over time in serum phosphorus (**A, B**) and alkaline phosphatase (**C,D**) among patients with normal and clinically normal limbs versus patients with varus/valgus limbs. For serum phosphorus, the dashed line indicates the lower limit of normal (3.2 mg/dL). For alkaline phosphatase, the dash line indicates 100% of the upper limit of normal based on age and sex, calculated from the following normal ranges: girls aged 1 to 4 years, 317 U/L; girls aged 4 to 7 years, 297 U/L; girls aged 7 to 10 years, 325 U/L; girls aged 10 to 15 years, 300 U/L; boys aged 1 to 4 years, 383 U/L; boys aged 4 to 7 years, 345 U/L; boys aged 7 to 10 years, 309 U/L; and boys aged 10 to 15 years, 385 U/L. ALP, alkaline phosphatase; SD, standard deviation; ULN, upper limit of normal.
